# Carotid‐Type Eagle Syndrome: Successful Management With Transcervical Styloidectomy: A Case Report

**DOI:** 10.1002/ccr3.72190

**Published:** 2026-02-26

**Authors:** Cristhian Garcia, Jessica Cayambe, Ana Perez, Fernanda Zambonino, Mikaela Garcia, Eduardo Pilatuna, Paola Solis‐Pazmino

**Affiliations:** ^1^ Institute of Thyroid and Head and Neck Diseases (ITECC) Quito Ecuador; ^2^ Cancer de Tiroides en Latino America (CaTaLiNA) Quito Ecuador; ^3^ School of Medicine Universidad San Francisco de Quito Quito Ecuador; ^4^ Surgery Group of Los Angeles Los Angeles California USA

**Keywords:** carotid space, eagle syndrome, elongated styloid process, presyncope, styloidogenic syndrome, transcervical styloidectomy

## Abstract

Carotid‐type Eagle syndrome should be considered in patients with unexplained presyncope, particularly when symptoms are position‐dependent. High‐resolution CT is essential for identifying elongated styloid processes and their relationship to the carotid space. Transcervical styloidectomy provides excellent vascular exposure, enabling safe dissection, complete resection, and effective symptom resolution while minimizing neurovascular complications.

## 1 Introduction

Eagle syndrome is an uncommon clinical entity resulting from an elongated styloid process or ossified/calcified stylohyoid ligament that impinges on adjacent neurovascular structures [1]. The syndrome is typically categorized into two subtypes. The classic form presents with cervicofacial pain, odynophagia, dysphagia, or foreign body sensation in the throat, usually due to cranial nerve compression, most often involving the glossopharyngeal nerve [2]. In contrast, the vascular (carotid) form occurs when the styloid process compresses the internal or external carotid artery, which can manifest as headache, transient ischemic attacks, Horner syndrome, or, rarely, carotid dissection and stroke [3].

Diagnosis requires a high index of clinical suspicion, with physical examination findings such as palpation of the styloid process in the tonsillar fossa that reproduces the symptom [3]. Imaging, particularly CT or 3D‐CT, is crucial for confirming the diagnosis, enabling accurate measurement of styloid length, assessing spatial relationships, and informing surgical planning [4].

While conservative measures (analgesics, local anesthetic infiltration) may be effective in mild cases, surgical resection of the elongated styloid process remains the definitive treatment, particularly for patients with severe or refractory symptoms [5]. Surgical approaches include intraoral and transcervical techniques, with evidence demonstrating higher rates of symptom resolution compared with nonoperative management [3].

This case report aims to describe a patient with carotid‐type Eagle syndrome, highlighting the clinical presentation, imaging findings, surgical management via transcervical styloidectomy, and postoperative outcomes, and to emphasize the importance of considering this diagnosis in patients with unexplained cervicofacial pain or neurovascular symptoms.

## 2 Case History/Examination

A 33‐year‐old man presented with a six‐month history of persistent left‐sided cervical pain, described as dull and intermittently radiating to the temporal region. The pain was associated with intermittent headaches and occasional presyncopal episodes, particularly triggered by cervical rotation or extension. Symptoms had progressively worsened despite treatment with analgesics and physical therapy. The patient denied prior cervical trauma, head and neck surgery, cardiovascular disease, or systemic illness.

On physical examination, the patient appeared well and in no acute distress. Deep palpation of the left tonsillar fossa reproduced his cervical pain, and neck rotation elicited mild discomfort. Neurological examination was normal, with intact cranial nerves, preserved motor strength and sensation, and normal reflexes. No cervical masses were palpated, and oropharyngeal and craniofacial examinations were unremarkable.

**FIGURE 1:**
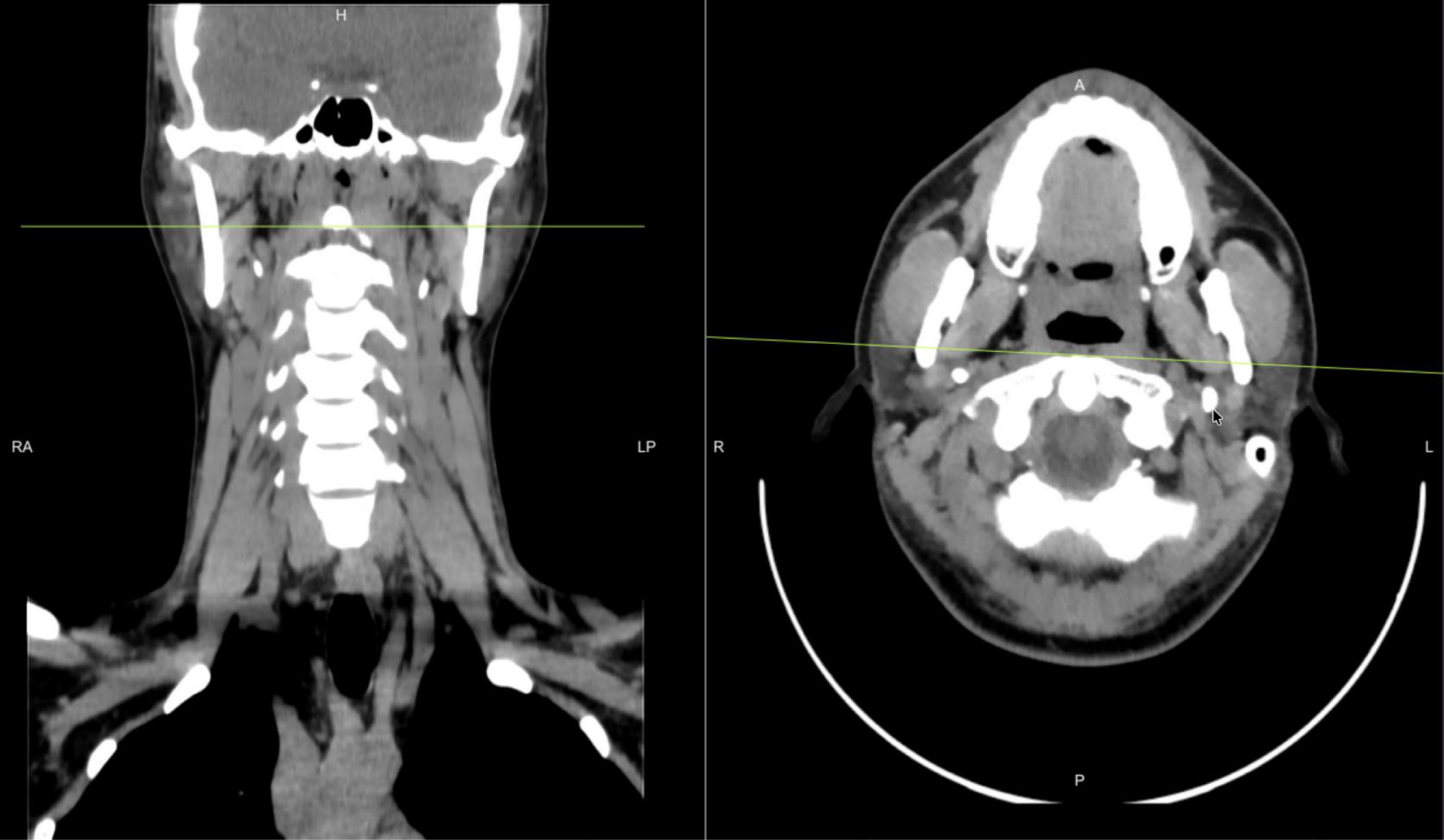
Non–contrast‐enhanced computed tomography (CT) of the neck with axial and coronal reconstructions (bone window) demonstrating bilateral elongated styloid processes.

**FIGURE 2:**
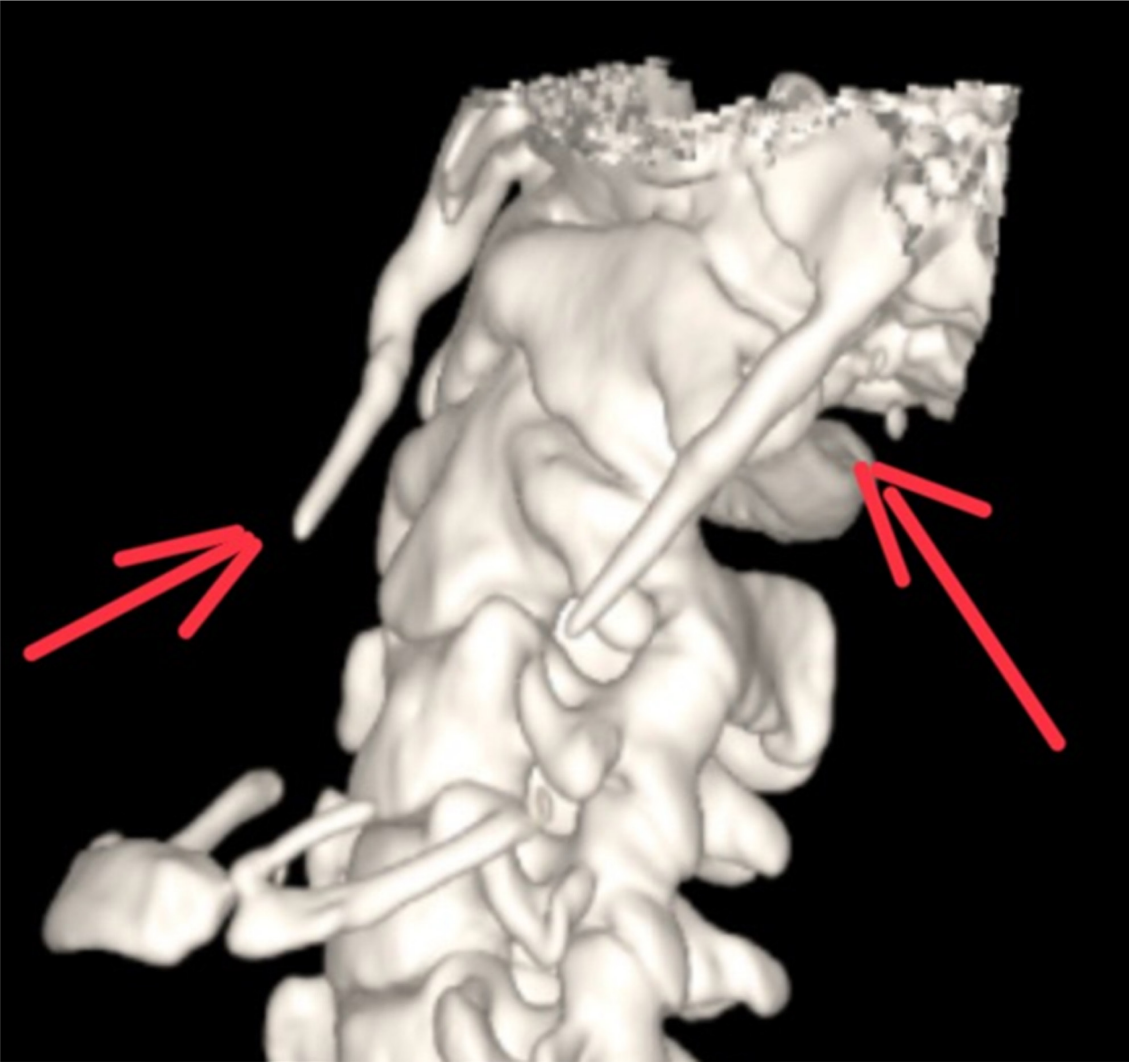
Three‐dimensional reconstructed CT image demonstrating bilateral elongated styloid processes projecting inferiorly, clearly visible on both sides of the skull. This finding supports the diagnosis of bilateral Eagle's syndrome.

Given the persistence and positional nature of symptoms, a non–contrast‐enhanced CT of the neck (Figure 1) with 3D volumetric reconstruction was performed. Imaging demonstrated bilateral elongation of the styloid processes, measuring 46 mm on the right and 48 mm on the left (Figure 2). On coronal and sagittal reconstructions, both styloid processes extended inferiorly from the temporal bone into the carotid space. The right styloid process was observed in close spatial proximity to the internal carotid artery, although no contrast‐enhanced imaging was available to demonstrate definitive vascular compression. No abnormalities were identified in the pharynx, larynx, cervical soft tissues, or vertebral alignment.

## 3 Differential Diagnosis and Management

Given the presence of presyncopal symptoms, which are not specific to styloidogenic syndromes, alternative causes were considered, including cardiovascular (arrhythmia, orthostatic hypotension), neurovascular, vestibular, cervicogenic, temporomandibular, and neuralgic etiologies. Clinical evaluation did not reveal evidence of cardiac or neurologic pathology, and symptoms were consistently reproduced by cervical movement and tonsillar fossa palpation.

Considering the severity of symptoms, the positional exacerbation, and the elongated styloid process in proximity to the carotid space, surgical management was pursued. A right‐sided transcervical (extraoral) styloidectomy was performed to allow optimal exposure of the carotid space and adjacent neurovascular structures.

## 4 Surgical Technique

**FIGURE 3:**
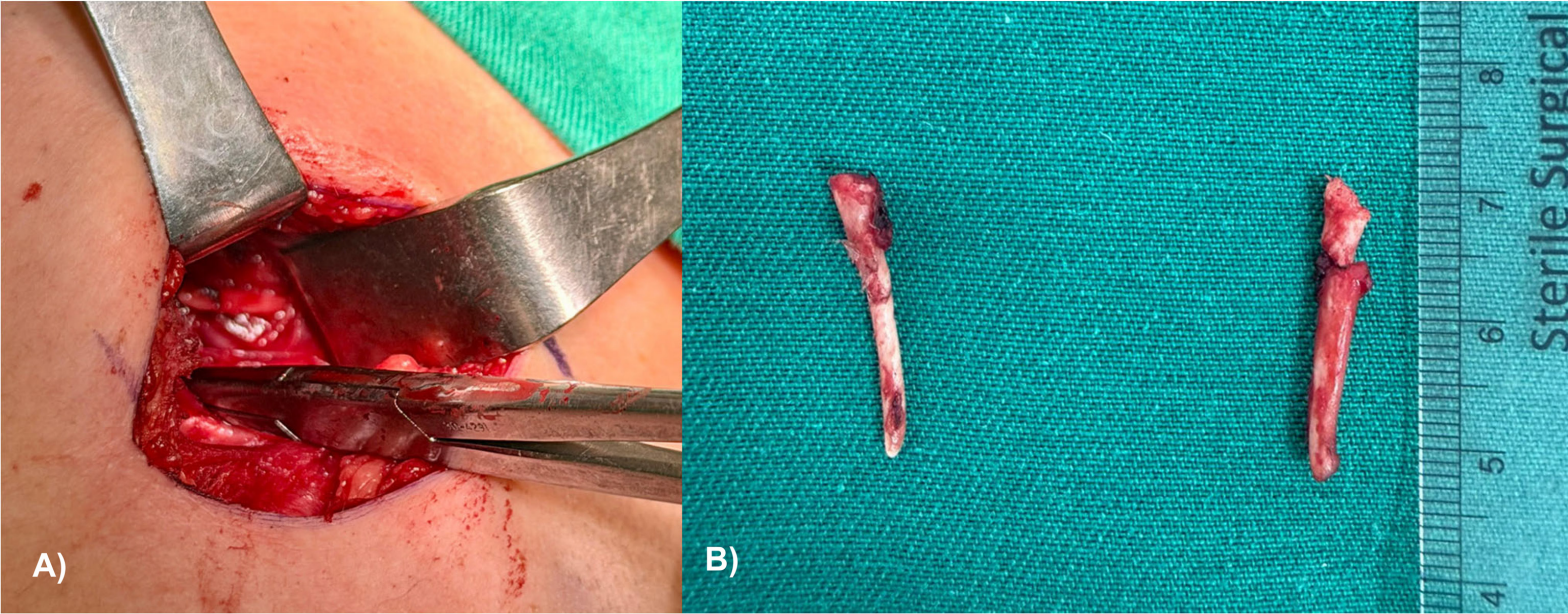
(A) Intraoperative view showing the elongated styloid process in proximity to adjacent anatomical structures prior to styloidectomy. The exposure demonstrates the spatial relationship of the styloid process to surrounding tissues, consistent with Eagle's syndrome findings. (B) Postoperative view demonstrating both styloid remnants after successful bilateral styloidectomy. The image clearly shows the resected ends (“horn‐like” projections) of the styloid processes following surgical removal.

A transcervical incision was made along a natural skin crease. After identification and retraction of the digastric muscle and stylohyoid complex, careful dissection was carried out to expose the elongated styloid process. The carotid sheath was identified and protected, and the surrounding cranial nerves were preserved. The styloid process was resected in a controlled fashion, ensuring complete removal while minimizing neurovascular risk (Figure 3A,B).

The procedure was completed without complications, and the patient was discharged on postoperative day three.

## 5 Follow‐Up

At 1‐, 3‐, and 6‐month follow‐up, the patient reported complete and sustained resolution of cervical pain, headaches, and presyncopal episodes. No postoperative complications were observed. Although the 6‐month follow‐up supports continued symptom relief, this duration remains limited, and longer‐term surveillance is recommended to assess durability and detect potential recurrence or reossification.

## 6 Discussion

Elongation of the styloid process or stylohyoid chain can produce a broad spectrum of cervicofacial and neurovascular symptoms [6]. In our case, the most relevant finding was the presence of marked bilateral styloid process elongation with a clear side‐to‐symptom correlation, in which the elongated right styloid process was spatially adjacent to the carotid space and reproduced the patient's position‐dependent presyncope. Although Eagle syndrome has traditionally been classified into “classic” and “vascular” subtypes, contemporary understanding increasingly supports a broader styloidogenic syndrome framework, in which symptoms arise from mechanical irritation of adjacent cranial nerves, vascular structures, or the pericarotid sympathetic plexus rather than from fixed vascular compression alone [7].

True vascular involvement requires objective demonstration of neurovascular conflict, ideally with contrast‐enhanced CT angiography [8]. In this case, non‐contrast CT confirmed significant bilateral elongation and demonstrated spatial proximity to the carotid space, which, when interpreted in conjunction with clinical findings, supported surgical intervention. However, we acknowledge that proximity alone does not confirm carotid compression, and our conclusions have been appropriately tempered.

The transcervical approach is particularly advantageous when elongated styloid processes extend deeply into the carotid space, as it provides superior visualization, vascular control, and reduced risk of incomplete resection compared with intraoral techniques [7]. Surgical resection remains the definitive treatment for symptomatic patients and is associated with high rates of symptom resolution [9].

Presyncope is an uncommon manifestation of styloid‐related syndromes and warrants careful evaluation for alternative etiologies. In the present case, symptom reproducibility with neck movement and local palpation, combined with imaging findings, supported a mechanical etiology.

## 7 Strengths and Limitations

A key strength of this case report is the detailed clinical–radiologic correlation in a patient with symptomatic elongated styloid processes, highlighting the importance of careful history, targeted physical examination, and high‐resolution CT with 3D reconstruction for diagnosis and surgical planning. The report also provides a stepwise description of the transcervical styloidectomy, emphasizing technical considerations and safety in the carotid space, with documented symptom resolution sustained at 6 months.

Several limitations should be acknowledged. As a single‐patient case report, the findings are not generalizable. In addition, contrast‐enhanced vascular imaging was not available, limiting definitive confirmation of carotid artery compression and necessitating cautious interpretation of vascular involvement. Although clinical improvement persisted at 6 months, longer follow‐up is required to assess long‐term durability and the risk of recurrence or reossification.

## 8 Conclusion

Elongated styloid process–related syndromes should be considered in patients presenting with unexplained cervical pain, headache, or position‐dependent presyncope. CT with 3D reconstruction is essential for confirming elongation and guiding surgical planning, while contrast‐enhanced imaging is required to definitively demonstrate vascular conflict. Transcervical styloidectomy offers a safe and effective treatment option for selected symptomatic patients. Longer‐term follow‐up is recommended to assess the durability of symptom relief.

## Author Contributions

**Cristhian Garcia:** conceptualization, data curation, formal analysis, funding acquisition, investigation, methodology, project administration, resources, software, supervision, validation, visualization, writing – original draft, writing – review and editing. **Jessica Cayambe:** data curation, funding acquisition, investigation, validation, writing – review and editing. **Ana Perez:** funding acquisition, investigation, validation, writing – review and editing. **Fernanda Zambonino:** funding acquisition, investigation, validation, writing – review and editing. **Mikaela Garcia:** funding acquisition, methodology, validation, writing – review and editing. **Eduardo Pilatuna:** funding acquisition, investigation, validation, writing – review and editing. **Paola Solis‐Pazmino:** conceptualization, data curation, formal analysis, funding acquisition, investigation, methodology, project administration, resources, software, supervision, validation, visualization, writing – original draft, writing – review and editing.

## Funding

The authors have nothing to report.

## Ethics Statement

This study was performed in line with the principles of the Declaration of Helsinki. The Ethics Committee of Universidad San Francisco de Quito approved.

## Consent

Written informed consent was obtained to publish this case report and accompanying images. On request, a copy of the written consent form is available for review by the editor‐in‐chief of this journal.

## Conflicts of Interest

The authors declare no conflicts of interest.

## Data Availability

The data that support the findings of this study are available on request from the corresponding author. The data are not publicly available due to privacy or ethical restrictions.

## References

[1] D. J. Fusco , S. Asteraki , and R. F. Spetzler , “Eagle's Syndrome: Embryology, Anatomy, and Clinical Management,” Acta Neurochirurgica 154, no. 7 (2012): 1119–1126, 10.1007/s00701-012-1385-2. 22638594

[2] S. Günaydın , Z. Memis , Ş. Kahraman , B. M. Yusumut , D. Şahmaran , and C. Uçan , “Bilateral Internal Carotid Artery Dissection as an Uncommon Complication Following a Fall in a Marathon Runner: A Case of Eagle Syndrome,” Radiology and Molecular Imaging (2024), 10.70087/rami.tui010107.

[3] G. Baldino , C. Di Girolamo , G. De Blasis , and A. Gori , “Eagle Syndrome and Internal Carotid Artery Dissection: Description of Five Cases Treated in Two Italian Institutions and Review of the Literature,” Annals of Vascular Surgery 67 (2020): 565.e17–565.e24, 10.1016/j.avsg.2020.02.033. 32205242

[4] A. Thielen , V. Brizzi , C. Majoufre , R. Nicot , and M. Schlund , “Eagle Syndrome and Vascular Complications—A Systematic Review,” International Journal of Oral and Maxillofacial Surgery 54, no. 1 (2025): 31–42, 10.1016/j.ijom.2024.09.011. 39406633

[5] H. Demirtaş , M. Kayan , H. R. Koyuncuoğlu , A. O. Çelik , M. Kara , and N. Şengeze , “Eagle Syndrome Causing Vascular Compression With Cervical Rotation: Case Report,” Polish Journal of Radiology 81 (2016): 277–280, 10.12659/PJR.896741. 27354882 PMC4912348

[6] J. Bargiel , M. Gontarz , K. Gąsiorowski , et al., “Stylohyoid Chain Syndrome (Eagle Syndrome) in Conjunction With Carotid Artery Dissection: A Case Report of Surgical Treatment,” Diseases 12, no. 1 (2024): 24, 10.3390/diseases12010024. 38248375 PMC10813943

[7] Z. Xu , P. Shi , and P. Zhang , “Eagle Syndrome: A Rare Neuropathic Disorder Affecting Head and Neck,” Medicine 103, no. 19 (2024): e38128, 10.1097/md.0000000000038128. 38728469 PMC11081567

[8] B. Schuurman , W. H. Mess , A. A. Postma , J. Staals , and S. van Weert , “Pearls & Oysters: Eagle Syndrome in a Patient With Neurologic Deficit Upon Head Rotation,” Neurology 105, no. 1 (2025): e213764, 10.1212/WNL.0000000000213764. 40456055

[9] S. Pagano , V. Ricciuti , F. Mancini , et al., “Eagle Syndrome: An Updated Review,” Surgical Neurology International 14 (2023): 389, 10.25259/sni_666_2023. 38053694 PMC10695462

